# Targeting Viral Ion Channels: A Promising Strategy to Curb SARS-CoV-2

**DOI:** 10.3390/ph15040396

**Published:** 2022-03-24

**Authors:** Anamika Singh, Isaiah T. Arkin

**Affiliations:** Department of Biological Chemistry, The Alexander Silberman Institute of Life Sciences, The Hebrew University of Jerusalem, Edmond J. Safra Campus, Jerusalem 9190400, Israel; anamika.iitr7@gmail.com

**Keywords:** COVID-19, ion channel, drug repurposing, antiviral drug, channel blocker

## Abstract

SARS-CoV-2 is the etiological agent COVID-19, one of the most impactful health crises afflicting humanity in recent decades. While research advances have yielded several treatment and prevention options, the pandemic is slow to abate, necessitating an expansion of our treatment arsenal. As a member of the coronaviridae, SARS-CoV-2 contains several ion channels, of which E and 3a are the best characterized. Since ion channels as a family are excellent drug targets, we sought to inhibit both viroporins as a means to curb infectivity. In a previous targeted study, we identified several blockers to each channel from an extensive drug repurposing library. Herein, we examined the ability of said compounds on the whole virus in cellulo. Gratifyingly, many of the blockers exhibited antiviral activity in a stringent assay examining protection from viral-driven death. In particular, darapladib and flumatinib, both 3a blockers, displayed potent antiviral activity. Furthermore, appreciable synergism between flumatinib and several E blockers was identified in a concentration regime in which the compounds are present in human plasma following oral administration. Taken together, targeting ion channels represents a promising approach to both augment and complement our antiviral arsenal against COVID-19.

## 1. Introduction

The COVID-19 pandemic emerged as a significant public health crisis since first detected in Wuhan, China [1,2,3,4]. The disease has a broad clinical spectrum in humans spanning from mild to severe manifestations, including lung injury and severe respiratory distress, with a mortality rate of about 1.4% worldwide [5].

Coronaviruses (CoVs) order Nidovirales, family Coronaviridae, subfamily Coronavirinae, are enveloped viruses with a positive sense, single-stranded RNA genome. SARS-CoV-2, the etiological agent of COVID-19 [2,3,6], belongs to the Sarbecovirus subgenus within the Betacoronavirus genus alongside its close homolog SARS-CoV-1 that caused the pandemic in 2002–2004 [7,8].

The immense impact of COVID-19 has brought about the development of treatment and prevention options at a record pace. Vaccines have proven particularly efficacious in preventing severe disease [9,10]. However, the constant emergence of new strains vitiates their ability to forestall infectivity [11].

Direct-acting antiviral drugs have also been developed against SARS-CoV-2, the first receiving emergency usage approval a handful of months after the pandemic broke [12,13]. However, later studies have questioned the efficacy of remdesivir as a viable option to mitigate COVID-19 [14]. Molnupiravir is another drug targeting the viral polymerase approved against SARS-CoV-2 [15]. More recently, a viral protease inhibitor was the third drug to receive approval [16].

Considering the speed at which new viral strains arise, it is imperative to continue developing new drugs, especially those targeting other virus proteins. To that end, we have decided to focus on the virus’s viroporins since, as a family, ion channels are excellent drug targets [17,18,19,20].

As a member of the coronaviridae, SARS-CoV-2 contains several viroporins, the best characterized of which are E and 3a. The E protein is the most conserved of all Sarbecoviruses’ proteins [6,21] and has been shown in other coronaviruses to have an essential role in virus production and maturation [22,23]. The Orf3a viroporin (3a) is involved in critical steps of the viral infection cycle and is required for viral replication and assembly that determines the virulence of SARS-CoV-2 [24,25,26]. Finally, efforts have culminated in the structural characterization of both channels [27,28].

Recently, we screened an extensive repurposed drug library for inhibitors against each channel using bacteria-based assays. Employing repurposed drugs can prove beneficial since the compounds’ toxicity is known while concomitantly focusing the chemical space for investigation. Our results yielded ten blockers identified against the 3a protein [29] and eight against E [30].

The present study reports the protective effect and antiviral activity of the aforementioned hits in a tissue culture setting. We demonstrate that flumatinib and darapladib, both 3a blockers, are particularly potent in preventing virus-induced cellular death. Additionally, several E channel blockers, such as mavorixafor and cyclen, exhibited marked synergism with the Flumatinib at low concentrations. Combinatorial regimens as such may improve efficacy and reduce the potential of the emergence of drug-resistant variants.

## 2. Results

The assay we chose to evaluate antiviral efficacy was protection from virus-induced cellular death. Precisely, the ability of every compound to enhance cellular viability was measured 48 h after infection and compared to untreated and uninfected cells.

### 2.1. Blocker Toxicity

**Figure A1:**
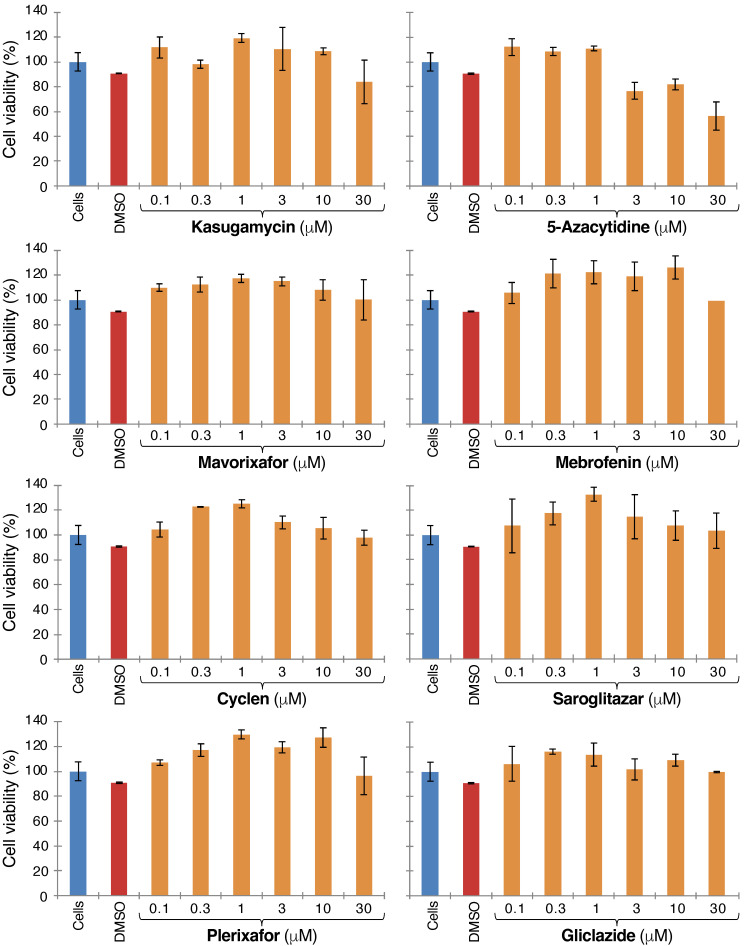
Toxicity of E channel blockers. Cellular viability after 48 h as a function of different compounds was monitored by MTS. Untreated cells were used as a comparison and normalized to 1.

**Figure A2:**
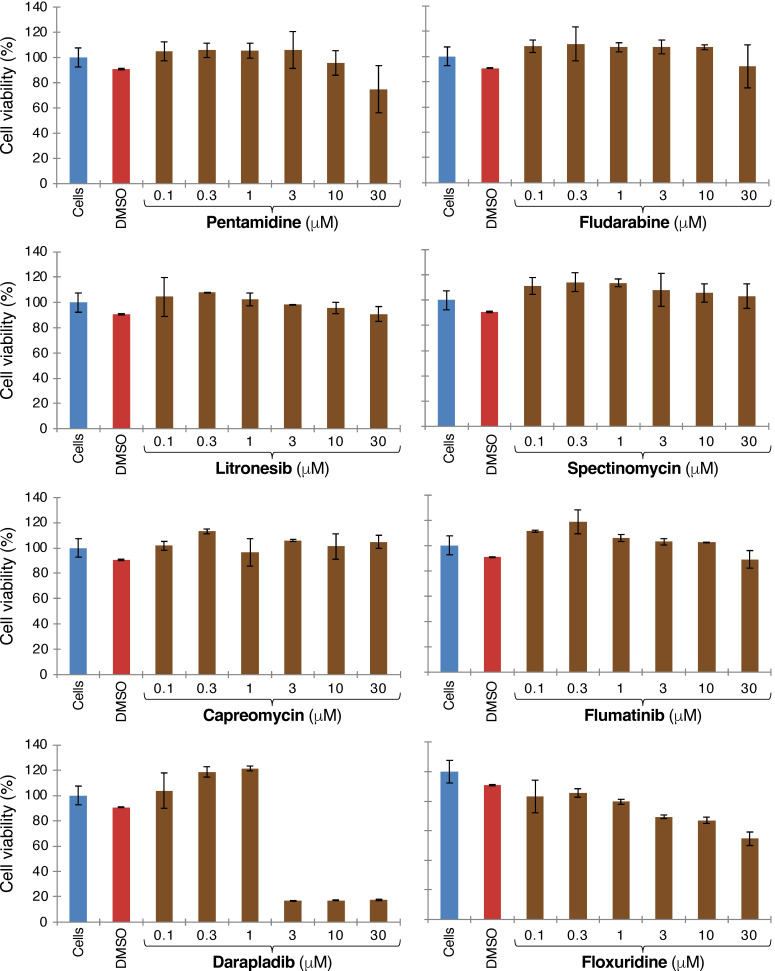
Toxicity of 3a channel blockers. Cellular viability after 48 h as a function of different compounds was monitored by MTS. Untreated cells were used as a comparison and normalized to 1.

Before analyzing the ability of compounds to protect cells from a viral infection, it was imperative to evaluate the inherent toxicity of every blocker. The reason being is that any toxic drug would score poorly in our assay even if it effectively inhibits a critical viral function. Moreover, tolerability studies as such provide a safe concentration range for investigation. Therefore, we tested cellular viability after 48 h in the presence of each drug at concentrations from 0.1–30 μM (Figure A1 and Figure A2). Results indicate that all drugs up to 30 μM did not affect cell viability appreciably upon comparison to the DMSO vehicle control, except for 5-azacytidine, darapladib, and floxuridine.

### 2.2. Antiviral Activity of Selected Blockers

We next assessed the potential of each drug to exert antiviral activity against SARS-CoV-2 using Vero E6 cells as an infection model [31]. Vero E6 cells were pretreated with an indicated amount of drugs for one hour, after which the cells were infected with a virus inoculum at a multiplicity of infection (MOI) of 0.01.

**Figure 1:**
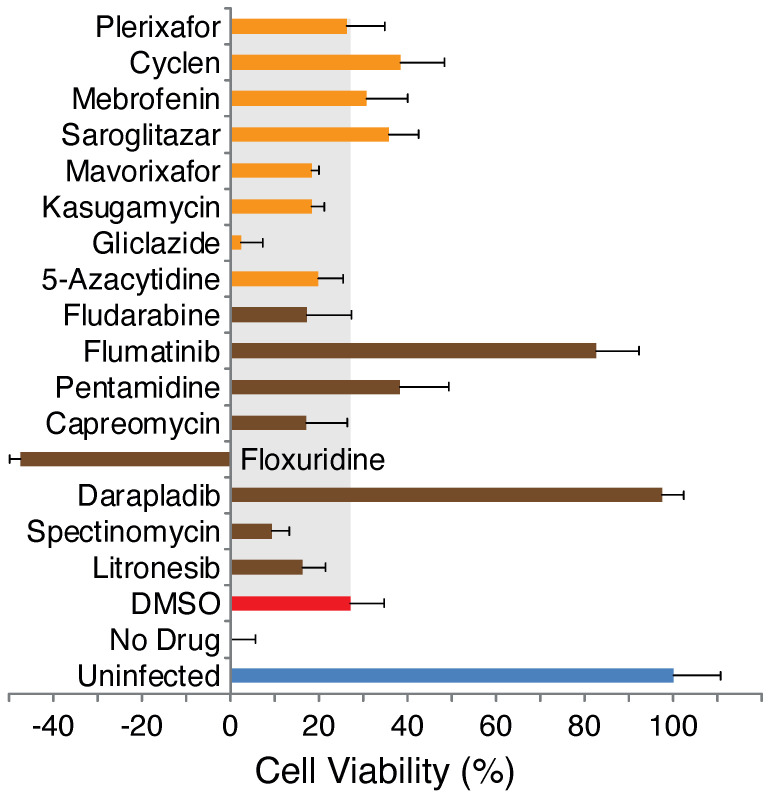
Antiviral activity of E and 3a blockers. Vero E6 cells were infected with an MOI of 0.01 and their viability is monitored by MTS after 48 h. At time 0, different drugs at a concentration of 3 μM are added (in 0.1% DMSO) as noted. Results are normalized relative to uninfected cells (100%) and untreated cells (0%). The gray region represents that the value of the mock treatment with 0.1% DMSO. Blockers of the E and 3a channels are shown in orange and brown, respectively.

**Figure 2:**
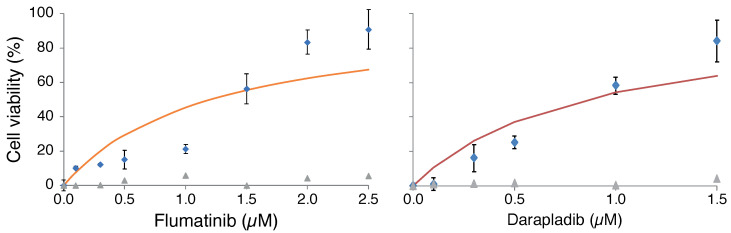
Dose-response analysis of darapladib and flumatinib. Vero E6 cells were infected with an MOI of 0.01 and their viability is monitored by MTS after 48 h. At time 0, darapladib (**right**) and flumatinib (**left**) at varying concentrations (in 0.1% DMSO) were added. Results are normalized relative to uninfected cells (100%) and untreated cells (0%). The solid line represents a best-fit using a one-term Monod equation (each blocker inhibits a single channel), while the residuals are shown in gray triangles.

**Figure A3:**
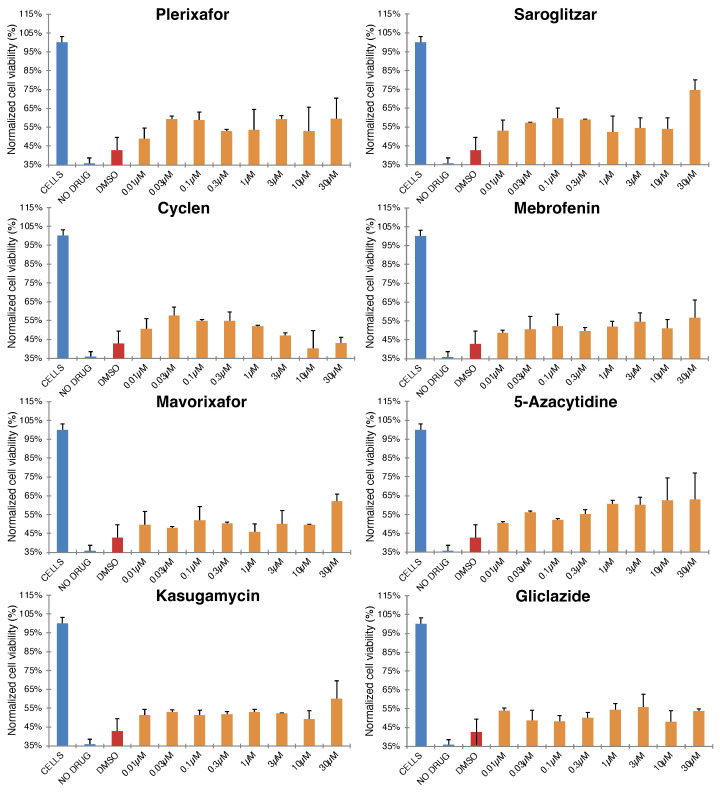
Dose-response analysis of E channel inhibitors. Vero E6 cells were infected with an MOI of 0.01 and their viability is monitored by MTS after 48 h. At time 0 the different compounds were added at varying concentrations (in 0.1% DMSO). Results are normalized relative to uninfected cells (100%).

**Figure A4:**
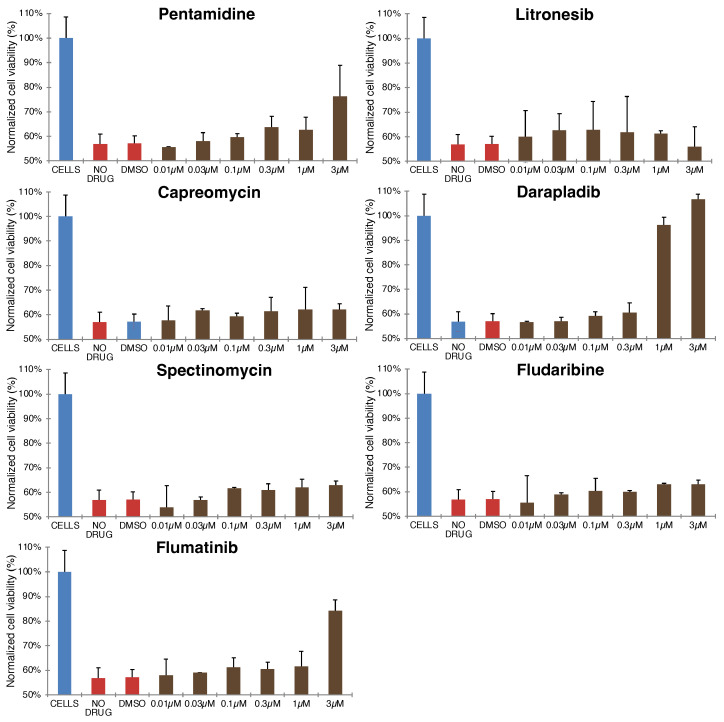
Dose-response analysis of 3a inhibitors. Vero E6 cells were infected with an MOI of 0.01 and their viability is monitored by MTS after 48 h. At time 0 the different compounds were added at varying concentrations (in 0.1% DMSO). Results are normalized relative to uninfected cells (100%).

As shown in Figure 1, several compounds exhibited significant protection from virus-induced cellular death at a concentration of 3 μM. In particular, darapladib and flumatinib, both 3a blockers, were markedly potent. Therefore, we followed by conducting a dose-response analysis of the two compounds as shown in Figure 2, yielding EC${}_{50}$ values of 0.4 μM and 1.6 μM for darapladib and flumatinib, respectively. Dose-response curves of all other drugs are presented in Figure A3 and Figure A4.

**Figure A5:**
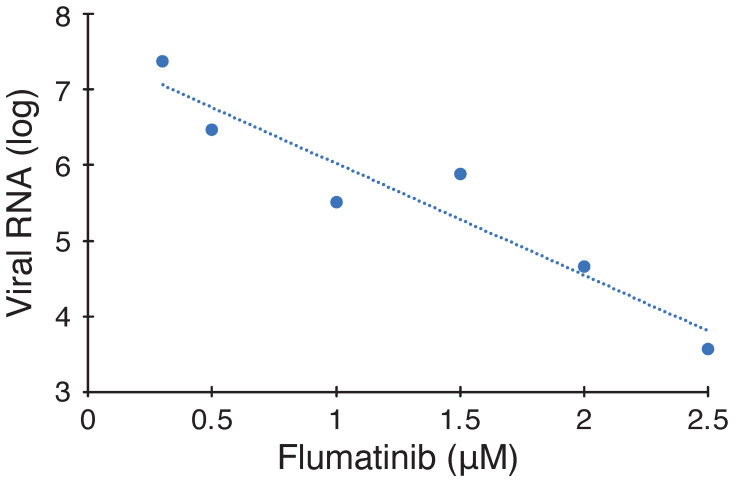
Flumatinib treatment reduces SARS-CoV-2 RNA levels in cell culture supernatant. Vero E6 cells were infected with an MOI of 0.01 and total RNA was extracted at 48 h post-infection. SARS-CoV-2 RNA was quantified by determining the amount of N gene using RT-qPCR.

To evaluate the stringency of the protection assay, we measured the resulting viral RNA levels as a function of different flumatinib concentrations (Figure A5). The result indicates that minor differences in viral protection lead to substantial differences in viral progeny. For example, the difference in the protection of flumatinib upon increasing the concentration from 0.3 μM to 1 μM is 26% (Figure 2). The exact concentration increase reduces viral RNA levels by 100 fold.

### 2.3. Drug Induced Phospholipidosis

**Figure A6:**
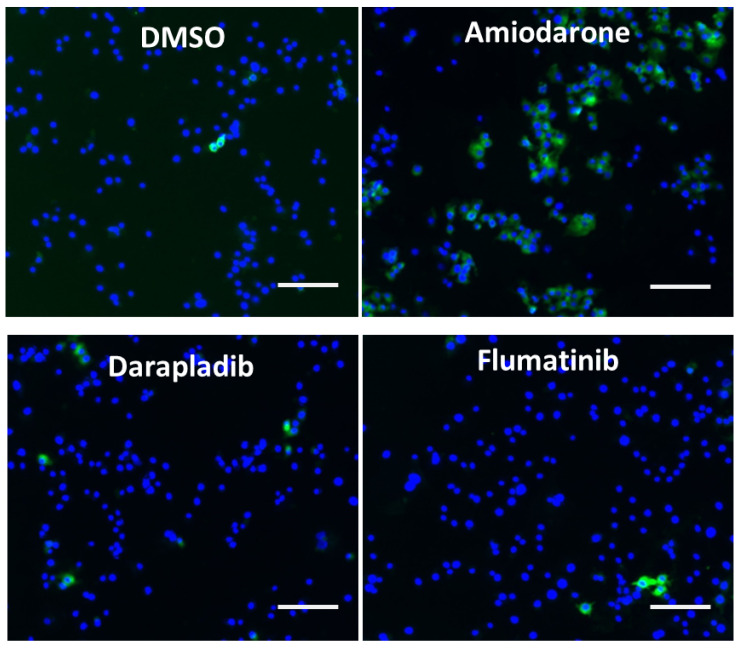
Phospholipidosis examination. Vero E6 cells were treated with 7.5 μM of the fluorescence phospholipid NBD-PE for 2 h. Subsequently, the cells were treated with darapladib (2 μM), flumatinib (3 μM), amiodarone (3 μM, as a positive control), and 0.1% DMSO (negative control) for 24 h. Fixation followed for 30 min with 3.7% formaldehyde and 2 μg/mL Hoechst 33258. Images were taken on a fluorescence microscope equipped with 10× and 40× objective and the LED/filter combinations DAPI and FITC for acquisition of Hoechst, NBD-PE dyes respectively. The bar size is 0.2 mm.

A recent study suggested that many cationic amphiphilic drugs indirectly exert their anti-SARS-CoV-2 activity via phospholipidosis induction [32]. Since darapladib and flumatinib can be classified as cationic amphiphiles, we examined their potential to induce phospholipidosis. The outcome of the analysis demonstrates that both drugs do not cause phsopholipidosis (Figure A6), and therefore, their activity is most likely against the virus directly.

### 2.4. Drug Synergism

**Figure 3:**
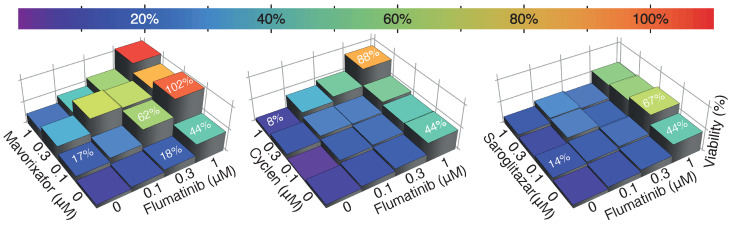
Synergism analyses between several E blockers and flumatinib. Cell viability after 48 h of infection (MOI 0.01) as a function of different combinations of drugs as noted. Results are normalized relative to uninfected cells (100%) and untreated cells (0%). Specific examples providing evidence for synergism are listed in the figure.

In order to examine potential synergism between the drugs, we tested the ability of different combinations to enhance viability. Particular synergistic effects were found between several E channel blockers and flumatinib, a 3a channel blocker (Figure 3). For example, flumatinib on its own at 0.3 μM provides 18% protection, and 0.1 μM mavorixafor on its own provides 17% protection. However, the combination of both drugs provides 62% protection. Similarly, flumatinib and cyclen, at 1 μM, offer 44% and 8% protection, respectively, while their combination offers 88% protection.

## 3. Discussion

The onset of the COVID-19 pandemic has spurred the rapid development of several prevention and treatment options. Vaccines have been particularly impactful at preventing severe disease [9,10]. However, the deterioration in their ability to reduce infectivity due to the emergence of new viral strains [11] motivates a search for additional treatment options.

Two proteins in the virus have received considerable attention as targets for pharmaceutical intervention: polymerase [15] and protease [16]. In order to bolster these efforts, we decided to focus on another functionality of the virus, its viroporins, and specifically, the E and 3a channels.

To that end, we have recently completed two extensive screens to identify inhibitors of the E [30] and 3a [29] channels of SARS-CoV-2. In brief, 2839 repurposed drugs were tested by three bacteria-based assays, yielding close to ten inhibitors of each channel. The goal of the current study was to examine if these blockers can inhibit the whole virus in a tissue culture setting.

We chose protection from virus-induced cell death from the several assays capable of investigating the antiviral activity. While it is a more stringent assay, protection from cellular death considers drug toxicity and does not necessitate additional controls. Furthermore, comparing protection from virus-induced death to measurements of viral progeny reduction demonstrates that the former is far more demanding: Even small changes in protection lead to considerable differences in viral RNA levels (Figure A5).

Analysis of all inhibitors at a single 3 μM dose indicated that several of the blockers are indeed capable of inhibiting virus-induced cellular death (Figure 1). Darapladib and flumatinib, both 3a inhibitors, particularly stood out, with EC${}_{50}$ values of 0.4 μM and 1.6 μM, respectively (Figure 2).

We recognize that both darapladib and flumatinib can be classified as cationic amphiphiles and have the potential to induce phospholipidosis. Moreover, a recent study showed that phospholipidosis might underpin the non-specific anti-SARS-CoV-2 activity of many cationic amphiphiles [32] Yet, both drugs’ examination demonstrates that they do not cause phsopholipidosis (Figure A6), thereby pointing to their direct antiviral activity.

It is important to note that the targeted screen that retrieved the compounds was conducted at a significantly higher concentration of 100 μM [29,30]. Therefore, it is no surprise that several of the inhibitors were unable to exhibit antiviral activity at 3 μM. However, employing a high screening concentration to cast a large net in the original screen was justified by the results of the synergism experiments. In particular, several of the inhibitors of the E channel exhibited synergism with flumatinib, an inhibitor of the 3a viroporin (Figure 3).

One synergism combination, flumatinib and mavorixafor, is particularly auspicious. Pharmacokinetic analyses following oral administration in humans demonstrate that both drugs are present in the plasma at the sub-microMolar concentrations in which pronounced synergism is obtained [33,34].

## 4. Materials and Methods

### 4.1. Inhibitors

A library of 2839 repurposed drugs was purchased from MedChem Express (HY-035, Monmouth Junction; NJ, USA). The drugs for this study were selected from the previously characterized bacteria-based assays against the SARS-CoV-2 E protein channel [30] and 3a channel [29].

### 4.2. Cell Culture

Simian kidney Vero E6 (ATCC Vero C 1008) cells were maintained in Dulbecco’s Modified Eagle Medium (DMEM); (Biological Industries; Beit Haemek, Israel ), supplemented with 10% fetal bovine serum, 2 mM L-Glutamine, 10 IU/mL Penicillin, and 10 μg/mL streptomycin (Biological Industries; Beit Haemek, Israel).

### 4.3. Virus Culture and Infection

SARS-CoV-2, isolate USA-WA1/2020, NR-52281 was deposited by the Center for Disease Control and Prevention and obtained through BEI Resources, NIAID, and NIH. The virus stock was prepared by infecting Vero E6 monolayers with SARS-CoV-2 isolate followed by harvest of culture supernatants three days post infection. The virus titers of cleared infected cells’ supernatants were determined by a standard TCID${}_{50}$ assay on Vero E6 cells using confluent cells in 96-well microtiter plates and were stored at −80${}^{\circ }$. Subsequent infection of Vero E6 cells was carried out in DMEM containing 2% FCS (Biological Industries; Beit Haemek, Israel) and further incubated for 48 h at 37${}^{\circ }$ in a 5% CO${}_{2}$ atmosphere. All infection experiments were performed in a BSL-3 facility.

### 4.4. Compounds and Antiviral Screening Assay

A 10 mM stock of listed compounds was prepared in DMSO and stored at −80${}^{\circ }$ in aliquots until further use. Vero-E6 cells were seeded in 96-well flat bottom plates in 200 μL of medium at a density of 10,000 cells per well and grown overnight. The dilutions of tested compounds were prepared in DMEM with 2% FCS and 100 μL was added to the cells.

The effects of the drugs on the metabolic activity of Vero E6 cells were assessed at 48 h post treatment using CellTiter 96 Aqueous Non-Radioactive Cell Proliferation reagent (Promega; Madison, WI, USA).

To examine the effect of different drugs, cells were pretreated with the specific compound for one hour followed by infection with SARS-CoV-2 at a multiplicity of infection (MOI) of 0.01. Each compound concentration was tested in triplicate and each assay plate contained the following controls: no cells (background control), cells treated with medium (mock infection for normalization), infected/untreated cells and infected/solvent-treated cells (infection control).

At two days post infection, drug efficacies in control of toxicity were assessed by the CellTiter 96 Aqueous Non-Radioactive Cell Proliferation reagent (Promega; Madison, Wisconsin, United States) for 3 h at 37${}^{\circ }$ in a 5% CO${}_{2}$ atmosphere. Reactions were stopped and the virus inactivated by adding 30 μM of 4% formaldehyde. Absorbance was measured at 492 nm using a Tecan plate reader (Männedorf, Switzerland). Finally, the data were normalized to the mock-infected control, after which EC${}_{50}$ values were calculated by fitting the data to a Monod equation.

## 5. Conclusions

We have presented a targeted three-tier approach to identify anti-SARS-CoV-2 drugs. We started by identifying channels in the virus that may serve as drug targets. We continued by screening inhibitors against these viroporins utilizing a rapid bacteria-based methodology. We concluded by examining the ability of each of the hits to exhibit antiviral activity in tissue culture cells. Encouragingly, our approach was able to identify new anti-SARS-CoV-2 drugs that are attractive candidates for further development.

## Data Availability

Data is contained within the article.

## Appendix A. Materials and Methods

### Appendix A.1. RNA Extraction and Quantitative SARS-CoV2 RT-PCR

At two days post infection, the supernatants were collected for quantitative PCR with reverse transcription (RT–qPCR) to assess the viral load. Viral RNA was purified from the supernatants with the Aurum™ total RNA mini Kit (Bio-Rad, Hercules, CA, USA). Extracted viral RNA was stored at −80${}^{\circ }$ or used immediately for analysis by RT-PCR. The cDNA synthesis was performed according to the qScript cDNA synthesis kit protocol (Quanta bio, Beverly, MA, USA). Detection of SARS-CoV-2 RNA was performed with the primers sets specific to the nucleocapsid (N) gene. The Fwd 5${}^{\prime }$- CACCAAAAGATCACATTGGCACC-3${}^{\prime }$ and N-gene Rev 5${}^{\prime }$-GACTACGTGATGAGGAACGAGAAG-3${}^{\prime }$ primers were ordered from Integrated DNA Technologies (Coralville, IA, USA). A total of 2 μL of the cDNA prepared above was added per well of a 96-well plate. A mixture of 1× iTaq Universal SYBR green supermix (Bio-Rad, Hercules, CA, USA) and 200 nM forward and reverse detection primers was added to each well for a final volume of 20 μL.

As a standard, purified RNA of SARS-CoV-2 cell culture supernatant was used (stock concentration $1\times 10^{7}$ copies per mL) and cDNA was prepared and then a dilution series was prepared in RNA-elution buffer. Thermal cycle condition were adapted and modified from [35,36] Stage 1: 1 cycle at 95${}^{\circ }$ for 10 min, Stage 2: 45 cycles at 95${}^{\circ }$ for 15 s with 58${}^{\circ }$ for 30 s (CoV-2 N) and Stage 3: 1 cycle 95${}^{\circ }$ for 15 s, 60${}^{\circ }$ for 1 min and 95${}^{\circ }$ for 15 s using StepOnePlus™ Real-Time PCR System with StepOne™ Software Version 2.3 (Carlsbad, CA, USA). For each independent biological replicate, all qRT-PCR measurements, using N-gene primer pairs, were made in duplicate and all data sets included standard curves and negative/mock infected controls.

### Appendix A.2. Drug-Induced Phospholipidosis

The protocol used for the assay was implemented from the well-established nitrobenzoxadiazole conjugated phosphoethanolamine (NBD-PE) staining assay [32,37]. In brief, Vero E6 cells were cultivated in DMEM medium with 10% FCS, harvested and then seeded in a 12-well plate. A day after seeding, the cells were treated with 7.5 μM NBD-PE (Biotium, San Francisco, CA, USA) for 2 h at 37${}^{\circ }$. Subsequently, the cells were treated with 3μM of amiodarone (positive control), darapladib and flumatinib (tests) and 0.1% DMSO (negative control) for 24 h. After incubation for 24 h, the medium was gently aspirated, and cells were washed once with 37${}^{\circ }$ medium followed by fixation using PBS containing 3.7% formaldehyde and 2 μg/mL Hoechst 33258 (Sigma Aldrich, Rehovot, Israel). Fixation was allowed to proceed for 30 min at room temperature. Before Imaging cells were washed twice with the PBS and images were taken on a fluorescence microscope (Olympus, Tokyo, Japan) equipped with 10× and 40× objective and the LED/filter combinations DAPI and FITC for acquisition of Hoechst, NBD-PE dyes, respectively. The image analysis was performed using ImageJ software [38].
